# Hyperoxemia during resuscitation of trauma patients and increased intensive care unit length of stay: inverse probability of treatment weighting analysis

**DOI:** 10.1186/s13017-021-00363-2

**Published:** 2021-04-29

**Authors:** Ryo Yamamoto, Seitaro Fujishima, Junichi Sasaki, Satoshi Gando, Daizoh Saitoh, Atsushi Shiraishi, Shigeki Kushimoto, Hiroshi Ogura, Toshikazu Abe, Toshihiko Mayumi, Joji Kotani, Taka-aki Nakada, Yasukazu Shiino, Takehiko Tarui, Kohji Okamoto, Yuichiro Sakamoto, Shin-Ichiro Shiraishi, Kiyotsugu Takuma, Ryosuke Tsuruta, Tomohiko Masuno, Naoshi Takeyama, Norio Yamashita, Hiroto Ikeda, Masashi Ueyama, Toru Hifumi, Kazuma Yamakawa, Akiyoshi Hagiwara, Yasuhiro Otomo, on behalf of the Japanese Association for Acute Medicine (JAAM) Focused Outcomes Research in Emergency Care in Acute Respiratory Distress Syndrome, Sepsis and Trauma (FORECAST) Study Group

**Affiliations:** 1 Department of Emergency and Critical Care Medicine, Keio University School of Medicine, Tokyo, Japan; 2 Center for General Medicine Education, Keio University School of Medicine, 35 Shinanomachi, Shinjuku, Tokyo, 160-8582 Japan; 3 Department of Acute and Critical Care Medicine, Sapporo Higashi Tokushukai Hospital, Sapporo, Japan; 4 Division of Acute and Critical Care Medicine, Department of Anesthesiology and Critical Care Medicine, Hokkaido University Graduate School of Medicine, Sapporo, Japan; 5 Division of Traumatology, Research Institute, National Defense Medical College, Tokorozawa, Japan; 6 Emergency and Trauma Center, Kameda Medical Center, Kameda, Japan; 7 Division of Emergency and Critical Care Medicine, Tohoku University Graduate School of Medicine, Sendai, Japan; 8 Department of Traumatology and Acute Critical Medicine, Osaka University Graduate School of Medicine, Osaka, Japan; 9 Department of General Medicine, Juntendo University, Tokyo, Japan; 10 Health Services Research and Development Center, University of Tsukuba, Tsukuba, Japan; 11 Department of Emergency Medicine, School of Medicine, University of Occupational and Environmental Health, Kitakyushu, Japan; 12 Division of Disaster and Emergency Medicine, Department of Surgery Related, Kobe University Graduate School of Medicine, Kobe, Japan; 13 Department of Emergency and Critical Care Medicine, Chiba University Graduate School of Medicine, Chiba, Japan; 14 Department of Acute Medicine, Kawasaki Medical School, Kurashiki, Japan; 15 Department of Trauma and Critical Care Medicine, Kyorin University School of Medicine, Tokyo, Japan; 16 Department of Surgery, Center for Gastroenterology and Liver Disease, Kitakyushu City Yahata Hospital, Kitakyushu, Japan; 17 Emergency and Critical Care Medicine, Saga University Hospital, Saga, Japan; 18 Department of Emergency and Critical Care Medicine, Aizu Chuo Hospital, Aizuwakamatsu, Japan; 19 Emergency & Critical Care Center, Kawasaki Municipal Kawasaki Hospital, Kawasaki, Japan; 20 Advanced Medical Emergency & Critical Care Center, Yamaguchi University Hospital, Ube, Japan; 21 Department of Emergency and Critical Care Medicine, Nippon Medical School, Tokyo, Japan; 22 Advanced Critical Care Center, Aichi Medical University Hospital, Nagakute, Japan; 23 Advanced Emergency Medical Service Center, Kurume University Hospital, Kurume, Japan; 24 Department of Emergency Medicine, Teikyo University School of Medicine, Tokyo, Japan; 25 Department of Trauma, Critical Care Medicine, and Burn Center, Japan Community Healthcare Organization, Chukyo Hospital, Nagoya, Japan; 26 Department of Emergency and Critical Care Medicine, St. Luke’s International Hospital, Tokyo, Japan; 27 Division of Trauma and Surgical Critical Care, Osaka General Medical Center, Osaka, Japan; 28 Center Hospital of the National Center for Global Health and Medicine, Tokyo, Japan; 29 Trauma and Acute Critical Care Center, Medical Hospital, Tokyo Medical and Dental University, Tokyo, Japan

**Keywords:** Hyperoxemia, Hyperoxia, Trauma, Critically ill, ICU length of stay, Mortality

## Abstract

**Background:**

Information on hyperoxemia among patients with trauma has been limited, other than traumatic brain injuries. This study aimed to elucidate whether hyperoxemia during resuscitation of patients with trauma was associated with unfavorable outcomes.

**Methods:**

A post hoc analysis of a prospective observational study was carried out at 39 tertiary hospitals in 2016–2018 in adult patients with trauma and injury severity score (ISS) of > 15. Hyperoxemia during resuscitation was defined as PaO_2_ of ≥ 300 mmHg on hospital arrival and/or 3 h after arrival. Intensive care unit (ICU)-free days were compared between patients with and without hyperoxemia. An inverse probability of treatment weighting (IPW) analysis was conducted to adjust patient characteristics including age, injury mechanism, comorbidities, vital signs on presentation, chest injury severity, and ISS. Analyses were stratified with intubation status at the emergency department (ED). The association between biomarkers and ICU length of stay were then analyzed with multivariate models.

**Results:**

Among 295 severely injured trauma patients registered, 240 were eligible for analysis. Patients in the hyperoxemia group (*n* = 58) had shorter ICU-free days than those in the non-hyperoxemia group [17 (10–21) vs 23 (16–26), *p* < 0.001]. IPW analysis revealed the association between hyperoxemia and prolonged ICU stay among patients not intubated at the ED [ICU-free days = 16 (12–22) vs 23 (19–26), *p* = 0.004], but not among those intubated at the ED [18 (9–20) vs 15 (8–23), *p* = 0.777]. In the hyperoxemia group, high inflammatory markers such as soluble RAGE and HMGB-1, as well as low lung-protective proteins such as surfactant protein D and Clara cell secretory protein, were associated with prolonged ICU stay.

**Conclusions:**

Hyperoxemia until 3 h after hospital arrival was associated with prolonged ICU stay among severely injured trauma patients not intubated at the ED.

**Trial registration:**

UMIN-CTR, UMIN000019588. Registered on November 15, 2015.

**Supplementary Information:**

The online version contains supplementary material available at 10.1186/s13017-021-00363-2.

## Background

Oxygen administration has a vital role in the management of critically ill patients [1, 2]. However, supraphysiological oxygen tension in the blood and/or tissue, hyperoxemia, has been reported to affect mortality and intensive care unit (ICU) length of stay in different diseases [1, 3–5], such as traumatic brain injury [6, 7], post-cardiac arrest syndrome [8, 9], and post-cardiac surgery [10]. Moreover, various studies revealed that unnecessarily high fraction of inspired oxygen (FiO_2_) was also associated with increased mortality of critically ill patients [11, 12], including sepsis [13, 14].

As several pathophysiological mechanisms behind harmful effects of hyperoxemia have been investigated, brain injury and pulmonary toxicity are emphasized as pivotal causes of unfavorable clinical outcomes in critically ill patient [15, 16]. Paradoxical reduction of oxygen delivery to the brain, due to vascular constriction and mitochondrial dysfunction, was observed in patients with traumatic/ischemic brain injury who experienced hyperoxemia [15, 17]. In addition, alveolar capillary injuries and pulmonary vasoconstriction inhibition by redundant reactive oxygen species with hyperoxemia was found in patients treated with mechanical ventilation [16, 18]. Furthermore, some basic studies suggested that hyperoxemia-induced acute lung injury (ALI) was exaggerated by inflammatory or lung-related biomarkers [19, 20].

Given that other subsets of critically ill patients, such as severely injured trauma patients, suffer from systematic inflammation, this population would be potentially affected by hyperoxemia. However, studies on clinical consequences of trauma patients who were exposed to hyperoxemia have been limited other than traumatic brain injury [21, 22]. Accordingly, this study aimed to elucidate whether hyperoxemia during resuscitation was associated with unfavorable clinical outcomes of trauma patients with severe injuries. We hypothesized that hyperoxemia exposure in the first 3 h after hospital arrival was associated with prolonged ICU stay. We also investigated the inflammatory and lung-protective biomarkers in patients with hyperoxemia to determine pathophysiological backgrounds of its potential harm.

## Methods

### Study design and settings

This study was a post hoc analysis of a nationwide multicenter prospective descriptive study conducted by the Japanese Association for Acute Medicine (JAAM) Focused Outcomes Research in Emergency Care in Acute Respiratory Distress Syndrome, Sepsis and Trauma (FORECAST) study group from April 1, 2016, to January 31, 2018. Patient data including blood samples were obtained from 39 emergency departments (EDs) and ICUs in tertiary hospitals [23]. The FORECAST TRAUMA study was registered at the University Hospital Medical Information Network Clinical Trial Registry on November 15, 2015 (UMIN-CTR ID, UMIN000019588). The JAAM approved this study, and all collaborating hospitals obtained approval of their individual institutional review board (IRB) for conducting research with human participants (approval number JAAM, 2014-01; approval number 014-0307 from Hokkaido University Graduate School of Medicine, Head institute of the FORECAST group; and approval number 20150056 from the Keio University School of Medicine Keio, institute of the corresponding author). This study was performed in accordance with the Declaration of Helsinki, and written informed consent was obtained from patients or their next of kin.

### Study population

The JAAM FORECAST TRAUMA study enrolled severely injured adult trauma patients (1) who were aged ≥ 16 years, (2) with injury severity score (ISS) of ≥ 16, and (3) who were directly transported from the scene. Patients without any available arterial partial pressure of oxygen (PaO_2_) data within 3 h after hospital arrival were excluded. The size of the study population was dependent on the study period.

### Data collection and definition

Patient data were prospectively collected and entered into an online data collection portal by treating physicians or volunteer registrars designated by each hospital. Available data included patient demographics, injury mechanism, vital signs on scene and hospital arrival, abbreviated injury scale (AIS), ISS, sequential organ failure assessment score, laboratory data including arterial blood gas and inflammatory and lung-related biomarkers (soluble receptor for advanced glycation end-products [sRAGE], high mobility group box-1 (HMGB-1), surfactant protein D [SPD], Clara cell secretory protein [CCSP], and interleukin-8 [IL-8]), amount of transfusion, resuscitative procedure conducted at the ED, any surgical procedures or angiography, ICU and hospital length of stay, and survival status at discharge.

Arterial blood gas was obtained on arrival and at 3 h post-admission without any prespecified exception, and hyperoxemia was defined as PaO_2_ of ≥ 300 mmHg. Hyperoxemia during resuscitation was defined as hyperoxemia on hospital arrival and/or at 3 h after admission. Inflammatory and lung-related biomarkers were obtained at the ED. The Charlson index was scored to assess comorbidities [24]. Isolated brain injury was defined as a head AIS of ≥ 3 and other regions of ≤ 1.

### Outcome measures

The primary outcome was ICU-free days until day 28, a composite of in-hospital mortality and ICU length of stay, defined as the number of days alive and out of the ICU between the day of hospital arrival and 28 days later. Secondary outcomes included survival to discharge and ventilator-free days until day 28.

### Statistical analysis

Patients were divided into hyperoxemia and non-hyperoxemia groups. The hyperoxemia group consisted of patients who experienced hyperoxemia during resuscitation (hyperoxemia on hospital arrival and/or at 3 h after admission), whereas the non-hyperoxemia group consisted of patients in whom hyperoxemia was not observed both on hospital arrival and at 3 h after admission. Considering that oxygen exposure during resuscitation and its pathophysiological effect on the pulmonary tissue would significantly differ between patient on mechanical ventilation and those who were not, analyses were performed on the whole population and those who were divided based on the intubation status at the ED. Unadjusted analysis was performed on the ICU-free days using the Mann–Whitney *U* test, and between-group differences were presented using the Hodges–Lehmann estimator of the median of all paired differences with 95% confidence intervals (CIs).

To adjust patient characteristics between the two groups, inverse probability of treatment weighting (IPW) analyses with propensity scores were performed to compare primary and secondary outcomes [25]. The propensity score was developed using the logistic regression model to estimate the probability of being assigned to the hyperoxemia group compared with the non-hyperoxemia group [26]. Relevant covariates were carefully selected from known or possible unfavorable clinical outcome predictors in trauma patients based on previous studies (such as age, comorbidities, injury mechanism, ISS, degree of chest injury, and requirement of tube thoracotomy), intubation status at the ED, and vital signs on hospital arrival. All of this information was subsequently entered into the propensity model [27–29], in which patients with missing covariates were excluded from the propensity score calculation. The precision of propensity score discrimination was analyzed using the c-statistic [26]. IPW analyses were then performed as adjusted analyses, in which primary and secondary outcomes were compared using Mann–Whitney *U* tests and chi-square tests [25]. IPW was performed with restriction, in which patient data with ≤ 0.1 or ≥ 0.9 of the propensity score were not used to avoid extreme weights. Between-group differences were presented using the Hodges–Lehmann estimator with 95% CIs.

Subgroup analyses were performed to further interpretate primary results. IPW analyses on the primary outcome were repeated after excluding patients who experienced hypoxia during resuscitation, defined as PaO_2_ of < 60 mmHg within 3 h of hospital arrival. Another subgroup analysis was conducted after excluding patients with persistent hyperoxemia, defined as PaO_2_ of ≥ 300 mmHg both on hospital arrival and at 3 h after admission. Moreover, subgroup analysis was performed after excluding patients with isolated brain injury.

Furthermore, to investigate pathophysiological backgrounds of potential harm of hyperoxemia, effects of inflammatory and lung-protective biomarkers on the ICU length of stay were evaluated among patients treated with hyperoxemia. Each biomarker was analyzed along with intubation status at the ED, using ordinal logistic regression analysis after adjustment by IPW.

Descriptive statistics are presented as median (interquartile range) or number (percentage) and compared using Mann–Whitney *U* tests, Chi-square tests, or Fisher’s exact tests, as appropriate. Missing/ambitious values were used without manipulation. To test for all hypotheses, a two-sided *α* threshold of 0.05 was considered statistically significant. All statistical analyses were conducted using the SPSS, version 26.0 (IBM, Armonk, NY, USA) and Microsoft Excel (Microsoft, Redmond, WA, USA).

## Results

**Fig. 1:**
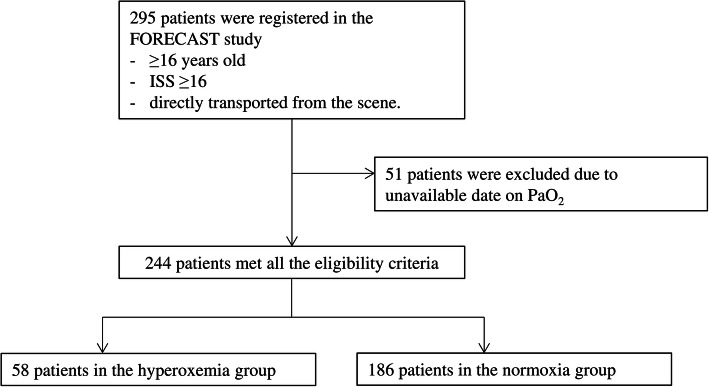
Patient flow diagram. A total of 295 patients with severe injuries were registered in the JAAM FORECAST TRAUMA study, which enrolled patients (1) aged ≥ 16 years, (2) with injury severity score (ISS) of ≥ 16, and (3) directly transported from the scene. Among them, 244 with available PaO_2_ within 3 h after hospital arrival were eligible for this study. Fifty-eight patients exposed to hyperoxemia (PaO_2_ ≥ 300 mmHg) within 3 h after arrival were included in the hyperoxemia group, whereas 186 not exposed to hyperoxemia were included in the non-hyperoxemia group

A total of 295 patients with severe injuries were registered in the JAAM FORECAST TRAUMA study. Among them, 244 with available PaO_2_ within 3 h of hospital arrival were eligible for this study. Figure 1 summarizes the patient flow diagram.

Fifty-eight patients were exposed to hyperoxemia (PaO_2_ of ≥ 300 mmHg) within 3 h of arrival and included in the hyperoxemia group, whereas 186 were not exposed to hyperoxemia and included in the non-hyperoxemia group. Table 1 summarizes patient characteristics. Compared with the non-hyperoxemia group, patients in the hyperoxemia group had lower Glasgow Coma Scale score [6 (3–13) vs 14 (11–15)] and higher ISS [29 (25–3) vs 26 (19–34)]. Furthermore, more patients in the hyperoxemia group required higher amount of blood products [red blood cell = 0 (0–4) vs 0 (0–2) and fresh frozen plasma = 0 (0–6) vs 0 (0–4)], had undergone craniotomy and angiography [19 (32.8%) vs 19 (10.5%) and 21 (36.2%) vs 37 (20.4%), respectively], and were intubated at the ED [47 (81.0%) vs 58 (31.2%)].

**Table 1:** Characteristics of patients with and without hyperoxemia

Case	Unadjusted			After IPW^a^		
	Hyperoxemia	Non-hyperoxemia	*P* value	Hyperoxemia	Non-hyperoxemia	*P* value
	58	186				
Age, years, median (IQR)	49 (35–67)	60 (46–75)	**0.022**	50 (31–65)	53 (37–69)	0.175
Sex, male, *n* (%)	34 (58.6%)	126 (67.7%)	0.202	83 (64.8%)	85 (65.9%)	0.860
Injury mechanism, blunt, *n* (%)	56 (98.2%)	180 (97.3%)	1.000	123 (97.6%)	125 (97.7%)	1.000
Comorbidities (Charlson index), median (IQR)	0 (0–0)	0 (0–0)	0.281	0 (0–0)	0 (0–0)	**0.007**
**Vital signs on arrival**, median (IQR)						
GCS	6 (3–13)	14 (11–15)	**< 0.001**	8 (5–14)	10 (6–14)	0.557
RR	21 (18–28)	21 (18–26)	0.687	20 (16–26)	20 (15–28)	0.696
HR	96 (77–120)	90 (73–102)	0.083	96 (80–120)	90 (73–110)	0.153
BP systolic, mmHg	124 (90–147)	129 (103–154)	0.353	133 (103-151)	123 (83–158)	0.220
**Injury severity**, median (IQR)						
AIS—head	4 (0–5)	2 (0–4)	**< 0.001**	4 (0–5)	4 (0–5)	0.663
AIS—chest	3 (0–4)	3 (0–4)	**0.039**	3 (0–4)	3 (0–4)	0.136
ISS	29 (25–38)	26 (19–34)	**0.001**	29 (25–38)	29 (25–38)	0.352
SOFA score	10 (8–11)	8 (6–10)	**0.001**	9 (7–11)	8 (7–11)	0.703
Cardiac arrest after arrival, *n* (%)	1 (1.7%)	2 (1.1%)	0.559	2 (1.6%)	4 (3.1%)	0.684
**Treatment**						
Tube thoracotomy, *n* (%)	13 (22.4%)	44 (23.7%)	0.845	32 (25.0%)	40 (31.0%)	0.284
Intubation at ED, *n* (%)	47 (81.0%)	58 (31.2%)	**< 0.001**	90 (70.3%)	96 (74.4%)	0.462
Transfusion^b^, *U*, median (IQR)						
RBC	0 (0–4)	0 (0–2)	**0.017**	0 (0–4)	0 (0–6)	0.230
FFP	0 (0–6)	0 (0–4)	**0.024**	0 (0–4)	0 (0–6)	0.218
Platelet	0 (0–0)	0 (0–0)	0.276	0 (0–0)	0 (0–0)	0.178
**Hemostatic procedure**, *n* (%)						
Craniotomy	19 (32.8%)	19 (10.5%)	**< 0.001**	37 (28.9%)	29 (23.2%)	0.301
Thoracotomy	2 (3.4%)	2 (1.1%)	0.248	4 (3.1%)	1 (0.8%)	0.181
Laparotomy	6 (10.3%)	13 (7.2%)	0.438	11 (8.7%)	10 (8.0%)	0.849
Angiography	21 (36.2%)	37 (20.4%)	**0.015**	34 (26.6%)	32 (25.4%)	0.832

*IPW* inverse probability weighting, *GCS* Glasgow Coma Scale, *RR* respiratory rate, *HR* heart rate, *BP* blood pressure, *AIS* Abbreviated Injury Scale, *ISS* Injury Severity Score, *SOFA* sequential organ failure assessment, *ED* emergency department, *RBC* red blood cell, *FFP* flesh frozen plasma

^a^IPW was performed using propensity scores, and data were presented after excluding patients with propensity score of < 0.1 or > 0.9 ^b^Amount of transfusion was calculated until 3 h after hospital arrival

Arterial blood gas analyses and inflammatory biomarkers are shown in Table 2. PaO_2_, FiO_2_, and PaO_2_/FiO_2_ (P/F) ratio on hospital arrival and at 3 h after admission were higher among patients in the hyperoxemia group than those in the non-hyperoxemia group. The median PaCO_2_ on hospital arrival and at 3 h after admission was comparable between the two groups. Inflammatory and lung-protective biomarkers were available in 83 patients and also comparable between the two groups, except for HMGB-1 that was higher in patients with hyperoxemia than those without [20.6 (11.0–46.6) vs 13.7 (8.7–22.1)].

**Table 2:** Arterial blood gas and biomarkers in patients with hyperoxemia and non-hyperoxemia

	Hyperoxemia		Non-hyperoxemia		*P* value
Arterial blood gas on arrival, median (IQR)					
PaO_2_, mmHg	355	(304–413)	112	(78–192)	< 0.001
FiO_2_	1.0	(0.8–1.0)	0.6	(0.3–1.0)	< 0.001
P/F ratio, mmHg	389	(308–481)	251	(139–391)	< 0.001
PaCO_2_, mmHg	40	(36–44)	38	(34–44)	0.256
Arterial blood gas at 3 h, median (IQR)					
PaO_2_, mmHg	205	(127–311)	104	(82–163)	< 0.001
FiO_2_	0.5	(0.4–0.6)	0.4	(0.2–0.5)	< 0.001
P/F ratio, mmHg	424	(321–527)	344	(229–446)	< 0.001
PaCO_2_, mmHg	40	(37–45)	40	(35–45)	0.517
Biomarkers on arrival, median (IQR)					
sRAGE, pg/mL	1446	(596–2786)	1046	(724–2216)	0.530
HMGB-1, ng/mL	20.6	(11.0–46.6)	13.7	(8.7–22.1)	0.023
SPD, ng/mL	32.0	(17.2–47.6)	30.5	(17.4–47.6)	0.952
CCSP, ng/mL	13.6	(8.5–22.0)	9.2	(6.5–17.5)	0.086
IL-8, pg/mL	18.2	(8.0–29.4)	11.4	(8.0–29.8)	0.230

*P/F* PaO_2_/FiO_2_, *IQR* interquartile range, *sRAGE* soluble receptor for advanced glycation end-products, *HMGB-1* high mobility group box-1, *SPD* surfactant protein D, *CCSP* Clara cell secretory protein, *IL* interleukin

The propensity model predicting allocation to the hyperoxemia group was confirmed to have appropriate discrimination (c-statistic = 0.858), in which three patients were excluded due to missing covariates for the propensity score calculation. Patient characteristics after IPW are summarized in Table 1, in which most covariates were successfully adjusted.

ICU-free days were significantly fewer among patients exposed to hyperoxemia within 3 h after admission, compared with those not exposed to hyperoxemia in the unadjusted analysis [17 (10–21) vs 23 (16–26) days; difference in median = − 4 days (95% CI = − 2 to − 7 days); *p* < 0.001; Table 3]. IPW analysis revealed that hyperoxemia during resuscitation was significantly associated with prolonged ICU stay among patients not intubated at the ED [ICU-free days = 16 (12–22) vs 23 (19–26); median difference = − 5 (− 3 to − 10) days; *p* = 0.004], but not among those intubated at the ED [ICU-free days = 18 (9–20) vs 15 (8–23); median difference = 0 (− 3 to 3) days; *p* = 0.777]. IPW analysis also identified that hyperoxemia exposure during resuscitation was associated with fewer ventilator-free days among patients not intubated [25 (15–26) vs 28 (23–28); *p* = 0.014], but not among those intubated at the ED. Survival to discharge were comparable between the two groups.

**Table 3:** Hyperoxemia and ICU-free days

	Hyperoxemia	Non-hyperoxemia	Difference	95% CI	*P* value
Unadjusted analyses					
ICU-free days until day 28, median (IQR)	17 (10–21)	23 (16–26)	− 4	− 2 to − 7	**< 0.001**
- Intubated at ED	17 (11–20)	15 (8–23)	0	− 3 to 4	0.832
- Not intubated at ED	22 (10–28)	24 (21–27)	− 2	− 9 to 2	0.356
IPW^a^					
ICU-free days until day 28, median (IQR)	16 (10–22)	19 (12–24)	− 2	− 4 to 0	0.123
- Intubated at ED	18 (9–20)	15 (8–23)	0	− 3 to 3	0.777
- Not intubated at ED	16 (12–22)	23 (19–26)	− 5	−3 to − 10	**0.004**
Ventilator-free days until day 28, median (IQR)	19 (10–26)	22 (7–27)	− 1	− 3 to 0	0.123
- Intubated at ED	18 (0–20)	18 (4–25)	0	− 4 to 0	0.265
- Not intubated at ED	25 (15–26)	28 (23–28)	− 2	− 3 to 0	**0.014**
Survival rate	85.2%	82.7%	OR = 1.21	0.62 to 2.35	0.590

*CI* confidence interval, *IQR* interquartile range, *ED* emergency department, *IPW* inverse probability weighting

^a^IPW was performed using propensity scores, and data were presented after excluding patients with propensity score of < 0.1 or > 0.9

Subgroup analysis, excluding patients who experienced hypoxia (PaO_2_ of < 60 mmHg) within 3 h after hospital arrival, also revealed the association between hyperoxemia and prolonged ICU stay among patients not intubated at the ED [ICU-free days = 16 (12–22) vs 23 (20–26); median difference = − 5 (− 3 to − 10) days; *p* = 0.003; Table S1 (Additional file 1)]. Another subgroup analysis excluding patients with isolated brain injury revealed similar results [ICU-free days = 16 (12–22) vs 22 (19–26); median difference = − 5 (− 2 to − 10) days; *p* = 0.006; Table S1 (Additional file 1)]. Conversely, the analyses on patients without persistent hyperoxemia showed comparable ICU-free days between those with or without hyperoxemia on hospital arrival (ICU-free days = 16 (5–25) vs 22 (19–26); median difference = − 5 (− 16 to 3) days; *p* = 0.300).

**Fig. 2:**
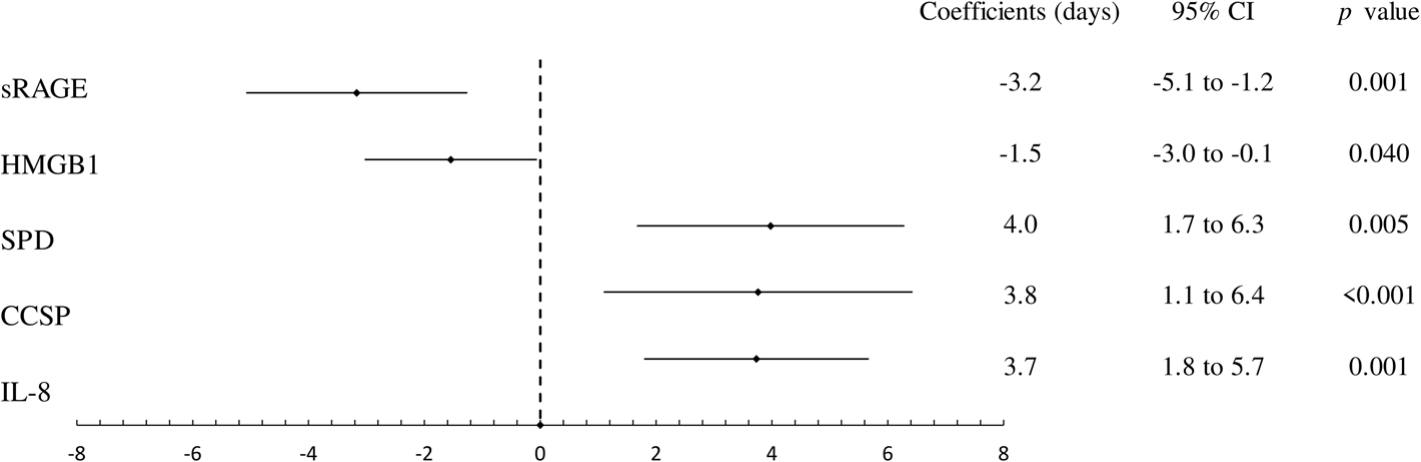
Effect of inflammatory and lung-related biomarkers on ICU-free days. Biological parameters were evaluated with multivariate regression among patients treated with hyperoxemia. CI, confidence interval; sRAGE, soluble receptor for advanced glycation end-products; HMGB-1, high mobility group box-1; SPD, surfactant protein D; CCSP, Clara cell secretory protein; IL, interleukin

Among patients in the hyperoxemia group, higher inflammatory biomarkers including sRAGE and HMGB-1 were associated with prolonged ICU stay [sRAGE (pg/mL), − 3.2 (− 5.1 to − 1.2) ICU-free days and HMGB-1 (ng/mL), − 1.5 (− 3.0 to − 0.1) ICU-free days; Fig. 2], whereas higher lung-protective proteins including SPD and CCSP were associated shorter ICU stay [SPD (ng/mL), 4.0 (1.7 to 6.3) ICU-free days and CCSP (ng/mL), 3.8 (1.1 to 6.4) ICU-free days; Fig. 2].

## Discussion

In these post hoc analyses of a nationwide multicenter prospective observational study, hyperoxemia during the initial resuscitation was found to be associated with prolonged ICU stay. This relationship was validated among patients not treated with mechanical ventilation at the ED, using IPW analyses that adjusted several prognostic factors. Notably, the observed association was consistent across several subgroup analyses.

Several reasons could be considered for the relationship between hyperoxemia exposure and prolonged ICU stay among severely injured trauma patients. First, hyperoxemia might have affected study participants who had moderate-to-severe traumatic brain injury. Although results on clinical consequences have been conflicting [30, 31], previous studies on traumatic brain injury reported that improvement of mitochondrial function in the injured cerebral tissue was not obtained by increasing FiO_2_ to 1.0 from 0.5. In addition, supranormal oxygen levels in the cerebral blood have been reported to suppress cell metabolism, resulting in paradoxical neuronal death [30, 32]. Second, supraphysiologic FiO_2_, hyperoxia, could induce ALI among considerable number of patients exposed to hyperoxemia. Several studies suggested that hyperoxia-induced ALI should be considered when FiO_2_ exceeds 0.6–0.7 and may become problematic when FiO_2_ exceeds 0.8 [33, 34]. In this study, the significantly higher FiO_2_ on hospital arrival was observed in the hyperoxemia group [1.0 (0.8–1.0)], and the higher FiO_2_ remained even at 3 h after admission. It should be also emphasized that the association between higher amount of lung-protective biomarkers and shorter ICU length of stay was observed in the hyperoxemia group, including CCSP, an important protein against oxidative stress in the respiratory system [35], and SPD, a pulmonary collectin against oxidative injury [36, 37].

Furthermore, systemic and/or lung tissue inflammation following severe injuries would have affected the baseline condition before hyperoxemia exposure. Given that animal studies found pre-administration of anti-inflammatory medication attenuated hyperoxia-induced ALI [38], systemic inflammation caused by trauma and/or chest injury itself would magnify the adverse effects of hyperoxia and hyperoxemia. Indeed, this study found that higher inflammatory biomarkers such as sRAGE and HMGB-1 were associated with unfavorable outcomes among patients exposed to hyperoxemia: sRAGE is a central cell surface receptor for HMGB-1, and both sRAGE and HMGB-1 are involved in the host response to injury, infection, and inflammation [39, 40].

Although a recent retrospective study on trauma patients reported that PaO_2_ of ≥ 150 mmHg on hospital admission was related to decreased in-hospital mortality, several differences should be noted in this study. First, the definition of hyperoxemia is different; patients with hyperoxemia (PaO_2_ of ≥ 150 mmHg) in the abovementioned study included only small number of patients exposed to PaO_2_ of ≥ 300 mmHg [median PaO_2_ was 230 (186–308) mmHg], although the harmful effect of hyperoxemia has been identified at PaO_2_ of ≥ 300 mmHg among critically ill patients [41, 42]. Second, hyperoxemia during resuscitation (on hospital arrival and at 3 h after admission) was examined in the current study because investigating only PaO_2_ on arrival would reflect prehospital treatment, rather than in-hospital critical care of trauma patients. Third, FiO_2_- and lung-related biomarkers were not measured in the abovementioned study, although hyperoxia-induced ALI has been suggested as a potential cause of unfavorable clinical outcomes in patients who experienced hyperoxemia [16, 18].

The harm of hyperoxemia during resuscitation was not confirmed in patients intubated at the ED in this study, probably because precise control over FiO_2_ during the lung-protective ventilation: Minimizing the length of exposure time to hyperoxia (supraphysiologic FiO_2_) would have diminished the relatively small degree of deleterious effects of hyperoxemia. The comparable mortality between the hyperoxemia and non-hyperoxemia groups obtained in this study is similar to that of a retrospective study on wartime pediatric trauma patients, which revealed no survival benefits of normoxia over hyperoxemia [21]. Considering that differences in the median ICU-free days between the two groups were only a few days in this study, prospective study involving sufficient number of patients should be conducted to confirm the possible harm of hyperoxemia in trauma patients.

The results of this study must be interpreted within the context of the study design. Post hoc analyses of the FORECAST TRAUMA study were conducted, which did not record indications of oxygen administration. Thus, our results could have been different if the respiratory condition during resuscitation had contained unrecorded strong prognostic factors. Another limitation is that variables relating to neurologic and pulmonary function were not available in the database. Although hyperoxemia-induced brain injury and ALI could be considered main causes of prolonged ICU stay following hyperoxemia exposure, objective data did not directly validate such physiological mechanism. Moreover, only hyperoxemia on hospital arrival and at 3 h after admission were investigated. Previous studies on hyperoxemia in various critically ill patients reported that clinical outcomes were different depending on timing (arrival, within a few hours, or within a day), definition (PaO_2_ ≥ 300 mmHg, ≥ 400 mmHg, or highest quartile of observed data), and obtained data (highest, lowest, or defined time point) for hyperoxemia [1, 9, 42]. Therefore, our results would vary if PaO_2_ was measured at different time points or if hyperoxemia was differently defined. Furthermore, this study was not designed to examine whether hyperoxemia would be more harmful than hypoxia. Considering that various studies reported the harmfulness of hypoxia during trauma resuscitation, hypoxia should be avoided more reliably. Finally, some biases could not be adjusted with IPW: some variables for propensity score calculation such as vital signs on arrival could be intermediate variables between prehospital hyperoxemia and outcomes, and survival bias would exist because hyperoxemia was defined based on PaO_2_ within 3 h after admission.

## Conclusions

This study identified that hyperoxemia within 3 h after hospital arrival was associated with prolonged ICU stay among severely injured trauma patients not intubated at the ED. Further research is necessary to elucidate the harmful effect of different degrees and durations of hyperoxemia exposure.

## Supplementary Information

**Additional file 1 **: **Table S1**. Hyperoxemia and ICU-free days in subgroups.

## Data Availability

The datasets analyzed during the current study are available from the corresponding author on reasonable request.

## Abbreviations

ICU: Intensive care unit
FiO_2_: Fraction of inspired oxygen
ALI: Acute lung injury
JAAM: Japanese Association for Acute Medicine
FORECAST: Focused Outcomes Research in Emergency Care in Acute Respiratory Distress Syndrome, Sepsis, and Trauma
ED: Emergency department
UMIN-CTR: University Hospital Medical Information Network Clinical Trial Registry
IRB: Institutional review board
ISS: Injury severity score
PaO_2_: Partial pressure of oxygen
AIS: Abbreviated injury scale
sRAGE: Soluble receptor for advanced glycation end-products
HMGB-1: High mobility group box-1
SPD: Surfactant protein D
CCSP: Clara cell secretory protein
IL-8: Interleukin-8
IPW: Inverse probability of treatment weighting
