# Supplementary material for: Hyperoxemia during resuscitation of trauma patients and increased intensive care unit length of stay: inverse probability of treatment weighting analysis
Source: World J Emerg Surg. 2021 Apr 29;16:19. doi: 10.1186/s13017-021-00363-2 (PMC8082221; doi:10.1186/s13017-021-00363-2)
Supplement: Supplementary file 1 — Additional file 1 : Table S1. Hyperoxemia and ICU-free days in subgroups. [file 13017_2021_363_MOESM1_ESM.docx]

| Table S1. Hyperoxemia and ICU-free days in subgroups^a^ | | | | | |  |
| --- | --- | --- | --- | --- | --- | --- |
|  | Hyperoxemia | Non-hyperoxemia | Difference | 95% CI | *P*-value |  |
| Without hypoxia (n = 223) | 16 (10–22) | 20 (13–24) | −2 | −5 to 0 | 0.044 |  |
| - Intubated at ED | 17 (8–21) | 15 (8–23) | −1 | −4 to 2 | 0.386 |  |
| - Not intubated at ED | 16 (12–22) | 23 (20–26) | −5 | −3 to -10 | 0.003 |  |
|  |  |  |  |  |  |  |
| Without persistent hyperoxemia (n = 227) | 17 (12–21) | 19 (12–24) | −2 | −5 to 2 | 0.291 |  |
| - Intubated at ED | 17 (12–21) | 15 (9–23) | 0 | −3 to 4 | 0.891 |  |
| - Not intubated at ED | 16 (5–25) | 22 (19–26) | −5 | −16 to 3 | 0.300 |  |
|  |  |  |  |  |  |  |
| Without isolated brain injury (n = 229) | 16 (10–22) | 19 (12–24) | −2 | −4 to 1 | 0.155 |  |
| - Intubated at ED | 18 (8–21) | 15 (8–23) | 0 | −3 to 3 | 0.768 |  |
| - Not intubated at ED | 16 (12–22) | 22 (19–26) | −5 | −2 to -10 | 0.006 |  |
| ^a^IPW was performed using propensity scores, and data were presented after excluding patients with propensity score of <0.1 or >0.9. Abbreviations = CI, confidence interval; ED, emergency department; and IPW, inverse probability weighting. | | | | | |  |
|  |  |  |  |  |  |  |
|  |  |  |  |  |  |  |
|  |  |  |  |  |  |  |
